# Draft Genome Sequence of the Radioresistant Bacterium *Deinococcus grandis*, Isolated from Freshwater Fish in Japan

**DOI:** 10.1128/genomeA.01631-15

**Published:** 2016-02-11

**Authors:** Katsuya Satoh, Takefumi Onodera, Kota Omoso, Kiyoko Takeda-Yano, Takeshi Katayama, Yutaka Oono, Issay Narumi

**Affiliations:** a Ion Beam Mutagenesis Research Group, Biotechnology and Medical Application Division, Quantum Beam Science Center, Japan Atomic Energy Agency, Takasaki, Gunma, Japan; b Cooperative Research Center of Life Sciences, Kobe Gakuin University, Kobe, Hyogo, Japan; c Graduate School of Life Sciences, Toyo University, Itakura, Gunma, Japan; d Faculty of Agriculture, Tokyo University of Agriculture and Technology, Fuchu, Tokyo, Japan; e Faculty of Human Development, Takasaki University of Health and Welfare, Takasaki, Gunma, Japan; f Faculty of Life Sciences, Toyo University, Itakura, Gunma, Japan

## Abstract

*Deinococcus grandis* is a radioresistant bacterium isolated from freshwater fish in Japan. Here we reported the draft genome sequence of *D. grandis* (4.1 Mb), which will be useful for elucidating the common principles of radioresistance in *Deinococcus* species through the comparative analysis of genomic sequences.

## GENOME ANNOUNCEMENT

Although the radioresistance of organisms varies greatly among species, there is a group of bacteria that shows extraordinary resistance to radiation. Members of the genus *Deinococcus* are the best known as radioresistant bacteria, and more than 50 *Deinococcus* species have been isolated from various environments (http://www.bacterio.net/deinococcus.html). Radioresistance in *Deinococcus* species is attributed to their highly proficient DNA repair capacity (1–4). *Deinococcus grandis* was initially isolated as a Gram-negative, red-pigmented, radioresistant, rod-shaped bacterium from freshwater fish and named *Deinobacter grandis* (5). Later, on the basis of 16S rRNA gene sequence analysis, *Deinobacter grandis* was transferred to the genus *Deinococcus* as *Deinococcus grandis* (6).

The draft genome sequence of *D. grandis* ATCC 43672 was 4,092,497 bp, with an average G+C content of 67.5%, and comprised 4 circular contigs (3,250,361 bp, 389,340 bp, 91,291 bp, and 8,055 bp) and 3 linear contigs (98,058 bp, 108,779 bp, and 146,613 bp). The linear contigs composed a single circular contig. This suggests that the genome structure of *D. grandis* is made up of multiple circular DNAs, as is the case in other *Deinococcus* species (*D. radiodurans*, *D. geothermalis*, *D. deserti*, *D. maricopensis*, *D. proteolyticus*, *D. gobiensis*, and *D. peraridilitoris*) (7). The sequences were obtained with the Roche GS Junior and Applied Biosystems 3500 genetic analyzers and assembled using the GS DeNovo assembler ver. 3.0 and DNASTAR SeqMan Pro ver. 12.2.0. Automatic annotation was performed using the Microbial Genome Annotation Pipeline (8), which predicted a total of 4,043 protein-coding sequences (CDSs). Moreover, all CDSs were manually validated. The tRNA and rRNA operon (5S/16S/23S) detections were performed using the tRNA scan software ver. 1.23 (9) and RNAmmer software ver. 1.2 (10), which predicted a total of 51 tRNAs and 4 rRNA operons, respectively.

The annotation of the draft genome sequence indicates that *D. grandis* possesses a DNA damage response regulator (encoded by a *pprI* homolog) and radiation-desiccation response (RDR) regulons (*recA*, *ddrA*, *ddrO*, *pprA*, and *gyrA* homologs, etc.), which are involved in the unique radiation/desiccation response system in *D. radiodurans* (4). *D. grandis* seems to employ the same radioresistance mechanisms as *D. radiodurans*. In future, the draft genome sequence of *D. grandis* will be useful for elucidating the common principles of radioresistance based on the extremely efficient DNA repair mechanisms in *Deinococcus* species through comparative analysis of genomic sequences. Furthermore, as the *D. grandis* host vector systems have already been developed (11), this genomic information will also be helpful for improvement of the host toward the efficient expression of endogenous and foreign genes.

### Nucleotide sequence accession numbers.

The draft genome sequence of *D. grandis* was deposited at DDBJ/EMBL/Genbank under the accession number BCMS00000000. The version described in this paper is the first version: BCMS00000000.1.
